# Thermoring basis for the TRPV3 bio-thermometer

**DOI:** 10.21203/rs.3.rs-2987105/v1

**Published:** 2023-06-02

**Authors:** Guangyu Wang

**Affiliations:** University of California, Davis

## Abstract

The thermosensitive transient receptor potential (TRP) channels are well-known as bio-thermometers with specific temperature thresholds and sensitivity. However, their structural origins are still mysterious. Here, graph theory was used to test how the temperature-dependent non-covalent interactions as identified in the 3D structures of thermo-gated TRPV3 could form a systematic fluidic grid-like mesh network with the thermal rings from the biggest grids to the smallest ones as necessary structural motifs for the variable temperature thresholds and sensitivity. The results showed that the heat-evoked melting of the biggest grids may control temperature thresholds to activate the channel while the smaller grids may act as thermo-stable anchors to secure the channel activity. Together, all the grids along the gating pathway may be necessary for the specific temperature sensitivity. Therefore, this grid thermodynamic model may provide an extensive structural basis for the thermo-gated TRP channels.

## Introduction

The thermosensitive transient receptor potential (TRP) channels are well known as biothermometers involving TRPV (vanilloid), TRPM (melastatin), TRPC (canonical), and TRPA (ankyrin). Their temperature thresholds (Tth) for activation range from noxious cold, cold, warm to noxious heat. Specifically, TRPV1 (> 42°C), TRPV2 (> 52°C), TRPV3 (> 32–39°C), TRPV4 (> 25–35°C), TRPM2, TRPM3, TRPM4, and TRPM5 are involved in warm to hot sensation. In contrast, TRPA1 (< 17°C) or TRPM8 (< 20–28°C) and TRPC5 (< 25–37°C) are sensitive to cold and cool temperatures. When compared to non-temperature-sensitive ones, they also have a high temperature sensitivity Q_10_ (the ratio of rates or open probabilities $\left(P_{o}\right)$ of an ion channel measured 10°C apart) [1–16]. However, the structural origins of the specific temperature thresholds and sensitivity are still known.

Of special interest, TRPV3, which is mainly expressed in skin keratinocytes and oral and nasal epithelia mediating thermal reception and pain sensation [5–6], undergoes sensitization together with TRPV2 while TRPV1 and 4 channels desensitize in response to successive heat stimuli [1, 7–8, 17–19]. Upon initial short heat stimulation within 100 ms, TRPV3 exhibits the high temperature threshold and sensitivity in the noxious temperature range above 50°C. After that intensive stimulation, it becomes responsive to warm temperatures with the low sensitivity. Further studies showed that the insertion of valine at position 412 dramatically eliminates the use-dependent heat sensitization of TRPV3 [19].

Following those findings, the primary cryo-electron microscopy (cryo-EM) structural studies indicated that TRPV3 is a homotetramer. Each monomer has S1-S6 as a transmembrane domain (TMD) and a large intracellular amino- (N-) terminal as an ankyrin repeat domain (ARD). S1-S4 form a voltage-sensor-like domain (VSLD) while S5-S6 and the pore helix and two pore loops are folded as a pore domain. Both the VSLD and the pore domain are swapped via a S4-S5 linker. The TRP helices, which are almost parallel to the membrane, interact with both the skirt ARD and the TMD. Several lipid sites were also found in their interfaces [20]. The pre-S1 domain, together with the carboxyl- (C-) terminal loop domain, couples the TMD with the ARD. The residues ^638^GLGD^641^ in the P-loop-extended region line the selectivity filter to permeate partially hydrated Na^+^, K^+^ or Ca^2+^ ions but not to function as an upper gate. In contrast, the narrowest pore constriction around M677 on S6 may act as a lower gate [20–21]. Although the state- and redox-dependent cryo-EM structures of mouse TRPV3 (mTRPV3) with or without the Y564A mutation at different temperatures are available [22–23], the specific structural motifs responsible for the use-dependent temperature threshold and sensitivity have not been pinpointed.

On the other hand, following the findings that a nucleic acid hairpin can function as a thermal ring with the number of H-bonds in the stem and the loop length to regulate the melting temperature threshold $\left(T_{m}\right)$ [24–26], a graph theory-based grid thermodynamic model has been developed to describe proteins as a systematic fluidic grid-like noncovalent interaction mesh network along a single polypeptide chain. Further, the $T_{m}$ of each grid and the grid-based systematic thermal instability $\left(T_{i}\right)$ have been defined and calculated and compared with relevant experimental values. In this way, the theoretical and experimental match allows the thermal rings from the biggest grid to the smallest one to be identified as the necessary structural motifs for the thermal stability and activity of globular proteins such as two classes of fructose aldolases from psychrophilic to mesophilic and hyperthermophilic [27–29]. In this regard, it is necessary to test if membrane proteins such as TRPV3 also use such a series of thermo-rings as necessary structural motifs to achieve the use-dependent thermal sensitization.

In this computational, graph theory was used to examine this hypothesis by carefully decrypting each grid in the grid-like non-covalently interacting mesh networks as identified in the cryo-EM structures of mTRPV3 with or without the Y564A mutation at different temperatures [22–23]. Once the biggest grid was identified, the calculated $T_{m}$ was compared with the experimental threshold. Further, the grid-based systematic thermal instability $\left(T_{i}\right)$ was also calculated as important energetic references to identify different gating states for the use-dependent sensitization. Finally, the systematic structural thermo-sensitivity (Ω_10_) between any two gating states was also calculated and compared with the experimental Q_10_ once defined as a heat-evoked change of the total chemical potential of all the grids upon a change in the total enthalpy included in non-covalent interactions along the same gating pathway of one subunit between two gating states within 10°C apart. Once all the three lines of calculated parameters were found to be close to the experimental ones of some redox- and lipid-dependent gating states, a closed and reduced state, a sensitized but oxidized state, and an open and oxidized state were identified with a reasonable energetic sequence for the use-dependent heat sensitization of TRPV3.

## Results

### Reduced and PC-free mTRPV3-Y564A had the biggest Grid _13_ in the Pre-S1/TRP interface for a calculated $T_{m}$ 38°C

The cryo-EM structures of both closed and open states in detergent-solubilized PC-free mTRPV3-Y564A were first sampled at 37°C after heat sensitization. Therefore, it is necessary to examine if the release of the phosphatidylcholine (PC) lipid from the vanilloid site by the Y546A mutation is responsible for the lower experimental temperature threshold and sensitivity [22].

**Figure 1:**
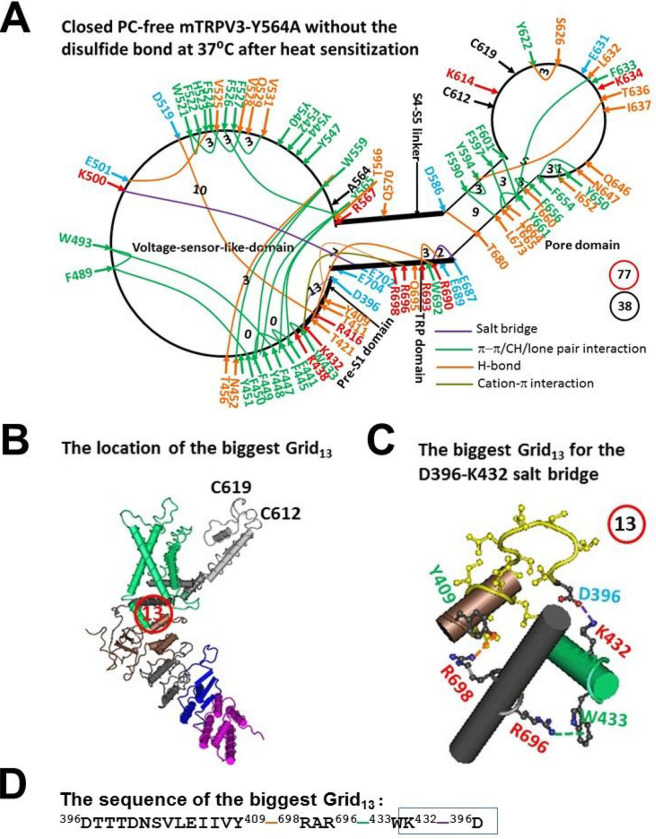
The grid-like non-covalently interacting mesh network along the gating pathway of PC-free reduced mTRPV3-Y564A in the sensitized state at 37 °C after heat sensitization. **A,** The topological grids in the systemic fluidic grid-like mesh network. The cryo-EM structure of one subunit in detergent-solubilzed mTRPV3-Y564A, which was PC-free, reduced, sensitized but closed at 37 °C (PDB ID, 6PVO), was used for the model. The pore domain, the S4-S5 linker, the TRP domain, the VSLD and the pre-S1 domain are indicated in black. Salt bridges, p interactions, and H-bonds between pairing amino acid side chains along the gating pathway from D396 to K705 are marked in purple, green, and orange, respectively. The grid sizes required to control the relevant non-covalent interactions were calculated with graph theory and labeled in black. The total grid sizes and grid size-controlled non-covalent interactions along the gating pathway are shown in the blue and black circles, respectively. **B,** The location of the biggest Grid_13_ is marked in a red circle. **C,** The structure of the biggest Grid_13_ with a 13-residue size in the TRP/pre S1 interface to control the D396-K432 salt bridge. **D,** The sequence of the biggest Grid_13_ to control the D396-K432 salt bridge in a blue rectangle.

The previous chimera studies between rat TRPV1 (rTRPV1) and mTRPV3 indicated that the pre-S1 segment 358–434 plays a critical role in mediating the temperature threshold and sensitivity Q_10_ [30]. On the other hand, the chimera investigations between heat-sensing TRPV1 and cold-sensing TRP melastatin 8 (TRPM8) showed that the C-terminal including the TRP domain (693–710) is required for the polarity of thermal sensitivity [31]. In this regard, the segment from D396 in the pre-S1 domain to K705 in the TRP domain should be at least included as the necessary gating pathway for the temperature threshold and sensitivity and the systemic thermal instability. Along such a gating pathway, the diversity of non-covalent interactions between amino acid side chains in the closed and PC-free Y564 mutant was found after heat sensitization (Fig. 1A). They included 9 H-bonds emerged between different hydrophilic residues, twenty-six π interactions between aromatic residues and nearby residues, and 3 salt bridges between several charged pairs (Fig. 1A, Table S1).

When these non-covalent interactions formed a systematic grid-like non-covalent interaction mesh network, the total non-covalent interactions and grid sizes along the gating pathway from D396 to K705 were 38 and 77, respectively (Fig. 1A). Thus, the systemic thermal instability $\left(T_{i}\right)$ was 2.03 (Table 1). Meanwhile, in addition to the smallest grid with a 0-residue size in the VSLD, the biggest Grid_13_ with a 13-residue size appeared in the pre-S1/TRP interface via the shortest path from D396 to Y409, R698, R696, W433, K432 and back to D396 to control the D396-K432 salt bridge (Fig. 1B–D). When 1.0 equivalent H-bond sealed this grid, the removal of the PC lipid from the vanilloid site by the Y564A mutation allowed a calculated $T_{m}$ of 38°C near the experimental $T_{m}$ 37°C (Table 1) [22].

### The melting of the biggest Grid_13_ at 37°C initiated channel opening of reduced and PC-free mTRPV3-Y564A with a low Ω_10_ comparable to the low Q_10_

**Figure 2:**
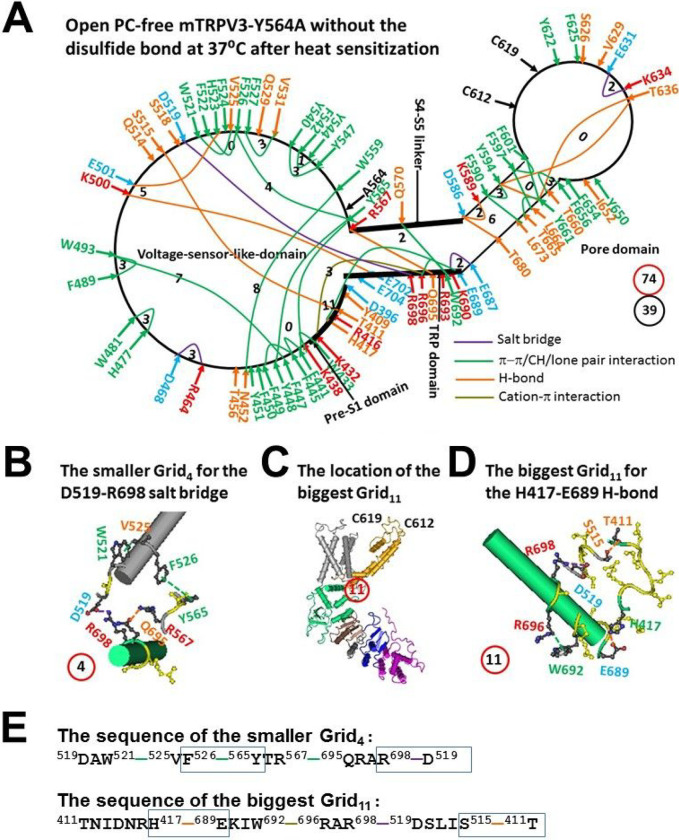
The grid-like non-covalently interacting mesh network along the gating pathway of PC-free reduced mTRPV3-Y564A in the open state at 37 °C after heat sensitization. **A,** The topological grids in the systemic fluidic grid-like mesh network. The cryo-EM structure of one subunit in detergent-solubilzed and open and reduced mTRPV3-Y564A without PC bound in cNW11 at 37 °C (PDB ID, 6PVP) was used for the model. The pore domain, the S4-S5 linker, the TRP domain, the VSLD and the pre-S1 domain are indicated in black. Salt bridges, p interactions, and H-bonds between pairing amino acid side chains along the gating pathway from D396 to K705 are marked in purple, green, and orange, respectively. The grid sizes required to control the relevant non-covalent interactions were calculated with graph theory and labeled in black. The total grid sizes and grid size-controlled non-covalent interactions along the gating pathway are shown in the blue and black circles, respectively. **B,** The structure of the smaller Grid_4_ with a 4-residue size to control the D519-R698 salt bridge and F526-Y565 p interaction in the grid. **C,** The location of the biggest Grid_11_ is marked in a red circle. **D,** The structure of the biggest Grid_11_ with an 11-residue size in the VSLD/TRP/pre S1 interfaces to control the H417-E689 and T411-S515 H-bonds. **E,** The sequences of two smaller Grid_4_ and Grid_11_ to control the aformentioned non-covalent interactions in the blue boxes, respectively.

When the mTRPV3-Y564A mutant opened with the melting of Grid_13_ in the TRP/pre-S1 interface at 37°C, the disruption of the D396-K432 salt bridge triggered several changes in the systematic grid-like non-covalent interaction mesh network. In addition to one salt bridge, three H-bonds and 17π interactions were conserved, two bridges, six H-bonds and eight π interactions were replaced with three new salt bridges, six new H-bonds and eight new π interactions (Tables S1 and S2). Thus, the total non-covalent interactions along the gating pathway from D396 to K705 had only a minor change from 38 to 39 (Figs. 1A and 2A).

On the other hand, the smallest Grid_0_ was conserved with a zero-residue size via the shortest path from F441 to Y565, Y448, F449, F445 and back to F441 (Figs. 1A and 2A). Thus, it may serve as a thermostable anchor against which two smaller grids may favor channel opening. One was Grid_4_ with a 4-residue size via the shortest path from D519 to W521, V525, F626, Y565, R567, Q695, R698 and back to D519 in the VSLD/TRP interface (Figs. 2A–B, E); the other was the biggest Grid_11_ in the TRP/VSLD/pre-S1 interfaces to control the H417-E689 π interaction. It had an 11-residue size via the shortest path from T411 to H417, E689, W692, R696, R698, D519, S515, and back to T411 (Figs. 2A, 2C–E). Once 2 equivalent H-bonds sealed the grid, the calculated $T_{m}$ was about 52°C (Table 1).

In any way, the disruption of the D396-K432 salt bridge in the biggest Grid_13_ induced a global conformational change from the pre-S1 domain to the VSLD, the TRP domain, the S4–55 linker and the pore domain (Figs. 1A and 2A). However, the grid sizes along the gating pathway from D396 to K705 had only a minor change from 77 to 74 (Figs. 1A and 2A). In this case, the systemic thermal instability $\left(T_{i}\right)$ was 1.90, and the calculated Ω_10_ was in a range from 0.76 to 4.30 and with a mean value 1.48, which was comparable to the experimental Q_10_ (~ 1.21) (Table 1) [22]. In other words, the removal of the PC lipid from the vanilloid site by the Y564A mutation allowed reduced mTRPV3 to have the very low structural and functional thermo-sensitivities.

On the other hand, oxidized mTRPV3 with a disulfide bond between C612 and C619 in the outer pore has also been reported to open from a PC-bound closed state at a lower threshold 42°C after repeated heat sensitization from 25°C to 40°C [23]. Therefore, it is exciting to test if oxidation also allows a low structural temperature sensitivity Ω_10_ to be responsible for the measured functional temperature sensitivity Q_10_ (1.9–3.1) [19].

### Closed PC-bound mTRPV3 with the disulfide bond in the outer pore had the biggest Grid17 in the Pre-S1/VSLD interface for a calculated $T_{m}$ 40°C after heat sensitization

**Figure 3:**
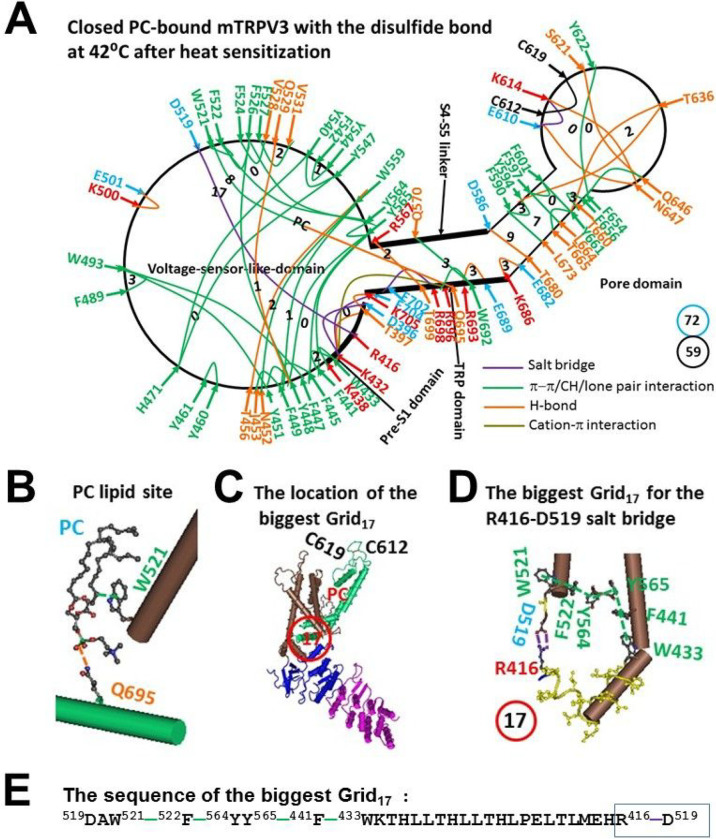
The grid-like non-covalently interacting mesh network along the gating pathway of PC-bound oxidized mTRPV3 in the sensitized state at 42 °C after heat sensitization. **A,** The topological grids in the systemic fluidic grid-like mesh network. The cryo-EM structure of one subunit in sensitized and oxidized mTRPV3 with PC bound in cNW11 at 42 °C (PDB ID, 7MIN) was used for the model. The pore domain, the S4-S5 linker, the TRP domain, the VSLD and the pre-S1 domain are indicated in black. Salt bridges, p interactions, and H-bonds between pairing amino acid side chains along the gating pathway from D396 to K705 are marked in purple, green, and orange, respectively. The grid sizes required to control the relevant non-covalent interactions were calculated with graph theory and labeled in black. The total grid sizes and grid size-controlled non-covalent interactions along the gating pathway from D396 to K705 are shown in the blue and black circles, respectively. **C,** The location of the biggest Grid_17_ is marked in a red circle. **D,** The structure of the biggest Grid_17_ with a 17-residue size in the VSLD/pre S1 interface to control the strong R416-D519 salt bridge. **E,** The sequence of the biggest Gird_17_ to control the R416-D519 salt bridge in a blue box.

Regarding the PC-bound closed state of oxidized mTRPV3 after heat sensitization, much more non covalent interactions than those in the PC-free closed state of reduced mTRPV3-Y564A shaped a distinct systematic fluidic grid-like non-covalent interaction mesh network (Figs. 1A and 3A). In the presence of the C612-C619 disulfide bond in the pore domain, four salt bridges (E610-K614 was merged into the C612-C619 disulfide bond), fifteen H-bonds and forty π interactions were identified between D396 and K705 (Fig. 3A, Table S3). Since the total non-covalent interactions and grid sizes along the gating pathway from D396 in the pre S1 domain to K705 in the TRP domain were 59 and 72, respectively (Fig. 3A), the grid-based systemic thermal instability $\left(T_{i}\right)$ was about 1.22 (Table 1). Despite several smallest grids with a zero-residue size, the biggest Grid_17_ with a 17-residue size was outstanding in the VSLD/pre-S1 interface to control the D519-R416 salt bridge (Fig. 3B–D). It started with D519 and went through W521, F522, Y564, Y565, F441, W433 and ended with R416 (Fig. 3E). When two equivalent H- bonds sealed the grid, the predicted $T_{m}$ was about 40°C (Table 1), which was close to the measured $T_{m}$ 42°C. [23]

### The melting of the biggest Grid_17_ at 42°C drove oxidized mTRPV3 opening with a low Ω_10_ comparable to the low Q_10_

**Figure 4:**
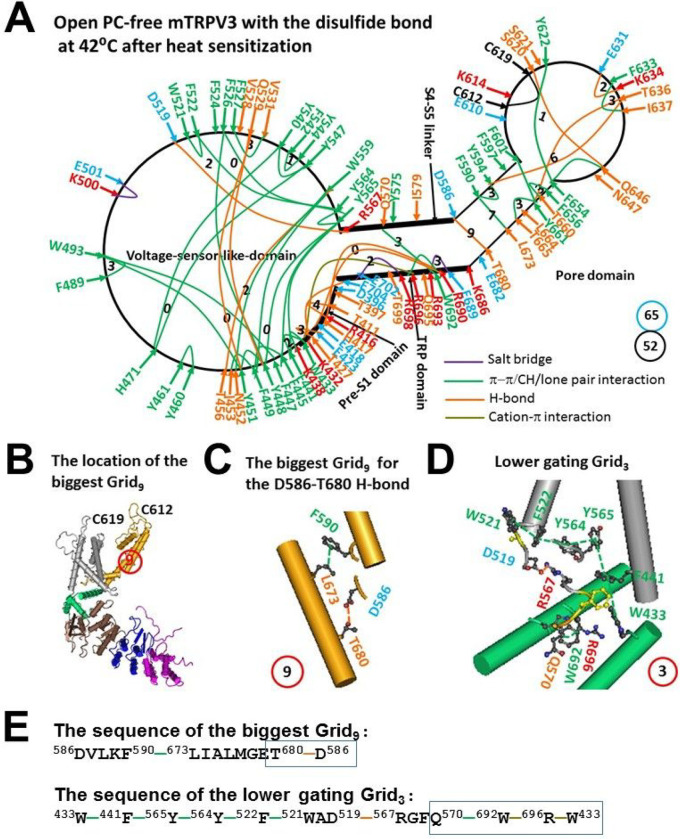
The grid-like non-covalently interacting mesh network along the gating pathway of PC-free oxidized mTRPV3 in the open state at 42 °C after heat sensitization. **A,** The topological grids in the systemic fluidic grid-like mesh network. The cryo-EM structure of one subunit in open and oxidized mTRPV3 without PC bound in cNW11 at 42 °C (PDB ID, 7MIO) was used for the model. The pore domain, the S4-S5 linker, the TRP domain, the VSLD and the pre-S1 domain are indicated in black. Salt bridges, p interactions, and H-bonds between pairing amino acid side chains along the gating pathway from D396 to K705 are marked in purple, green, and orange, respectively. The grid sizes required to control the relevant non-covalent interactions were calculated with graph theory and labeled in black. The total grid sizes and grid size-controlled non-covalent interactions along the gating pathway are shown in the blue and black circles, respectively. **B,** The location of the biggest Grid_9_ is marked in a red circle. **C,** The structure of the biggest Grid_9_ with a 9-residue size in the S5-S6 interface to control the D586-T680 H-bond. **D,** The structure of the putative smaller Grid_3_ with a 3-residue size for the lower gate. **E,** The sequences of two smaller gating Grid_9_ and Grid_3_ to control the D586-T680 H-bond and a group of crtitical cation-p interactions in the blue boxes, respectively.

In the heat-activated open state, following the melting of the R416-D517 salt bridge in the biggest Grid_17_ at 42°C as predicted (Fig. 3D) [23], although one salt bridge, five H-bonds, and 35π interactions were conserved, three salt bridges, ten H-bonds, and six π interactions were substituted by two new salt bridges, eight new H-bonds, and one new π interaction (Figs. 3A and 4A, Tables S3 and S4). However, two smallest Grid_0_ with a zero-residue size were still conserved as anchors near the R416-D519 salt bridge: one via the shortest path from F445 to Y565, Y448, F449, and back to F445, and the other via the shortest path from Y448 to Y565, Y564, F526 and back to Y448 (Figs. 3A and 4A). Therefore, the following gating pathway against these two anchors was proposed.

First, in the VSLD/pre-S1/TRP interfaces, the D519-R416 salt bridge was substituted by the T411-R416 and D519-R567 H-bonds. As a result, the T397-E704 H-bond and the D396-K432 salt bridge were broken with the formation of H417/E418-R690 and E423-T427 H-bonds. In addition, the K432-E704 salt bridge became an H-bond (Fig. 4A).

Second, when R567 H-bonded with D519 in the VSLD, a smaller Grid_2_ with a 2-residue size appeared via the shortest path from D519 to W521, F522, Y564, Y565, R567 and back to D519. Consequently, the V528-F524 π interaction was disconnected (Fig. 4A).

Third, when the conformational wave extended to the S4-S5/TRP interface, the R567-T699 H-bond was disrupted and the E689-R693 H-bond changed to a salt bridge (Fig. 4A).

Fourth, when this conformational wave continued to the pore domain, the F597-F601 π interaction and the N647-E610/K614 and E682-K686 H-bonds and the E610-K614 salt bridge were disconnected. In the meanwhile, the E631-K634 H-bond and the F633-I637 π interaction were present, and the H-bond moved from S621-Q646 to S620-Q646 (Fig. 4A).

Taking all these changes into account, after the biggest Grid_17_ in the VSLD/pre-S1 interface melted above the predicted 40°C, the PC lipid was released from nearby W521 and Q695 and thus the new biggest Grid_9_ with a 9-residue size was created in the S5-S6 interface, which may be required for channel opening (Fig. 4B–C). When two equivalent H-bonds sealed Grid_9_ via the shortest path from D586 to F590 and L673 and T680 and back to D586 (Figs. 4C, 4E), the calculated $T_{m}$ was about 56°C (Table 1). That may be why the temperature limit is 57°C for stable efficacy [19]. Since Grid_9_, together with Grid_7_ with a 7-residue size via the shortest path from F590 to Y594, T636, Y661, T665, L673 and back to F590, was conserved in both closed and open states of oxidized mTRPV3 (Figs. 3A and 4A), they may act as thermostable anchors to secure channel activity. In the meanwhile, a smaller Grid_3_ with a 3-residue size in the pre-S1/VSLD/S4-S5 linker/TRP/pre-S1 interfaces may be required to stimulate the lower state of the channel. It linked multiple active residues together including W433, F441, Y565, Y564, F522, W521, D519, R567, Q570, W692, and R696 (Figs. 4D, 4E).

As a result, the total non-covalent interactions and grid sizes along the gating pathway from D396 to K705 decreased from 59 and 72 to 52 and 65, respectively (Figs. 3A & 4A). Such a decrease produced a low systemic thermal instability $\left(T_{i}\right)$ value as 1.25. More importantly, the systematic structural thermo-sensitivity Ω_10_ was in a range from1.88 to 14.3 and with a mean value 4.12 (Table 1), which was close to the experimental Q_10_ (1.9–3.1) [19]. Therefore, even if the PC lipid at the corresponding vanilloid site was not released, the presence of the C612-C619 disulfide bond in the outer pore may be adequate for mTRPV3 to open with both low $T_{m}$ and Ω_10_ to match the measured T_th_ and Q_10_ in response to the second heat stimulation [19]. In that regard, it is attractive to test if the disruption of the C612-C619 disulfide bond can increase the $T_{m}$ and the Ω_10_ upon channel opening from reduced mTRPV3 to meet the requirement of the higher T_th_ (> 50 C) and Ω_10_ (16.4–22.6) [19].

### The melting of the biggest Grid12 at the vanilloid PC site above $T_{m}$ 50°C was required to release PC from reduced mTRPV3 for channel opening with a high Ω_10_

**Figure 5:**
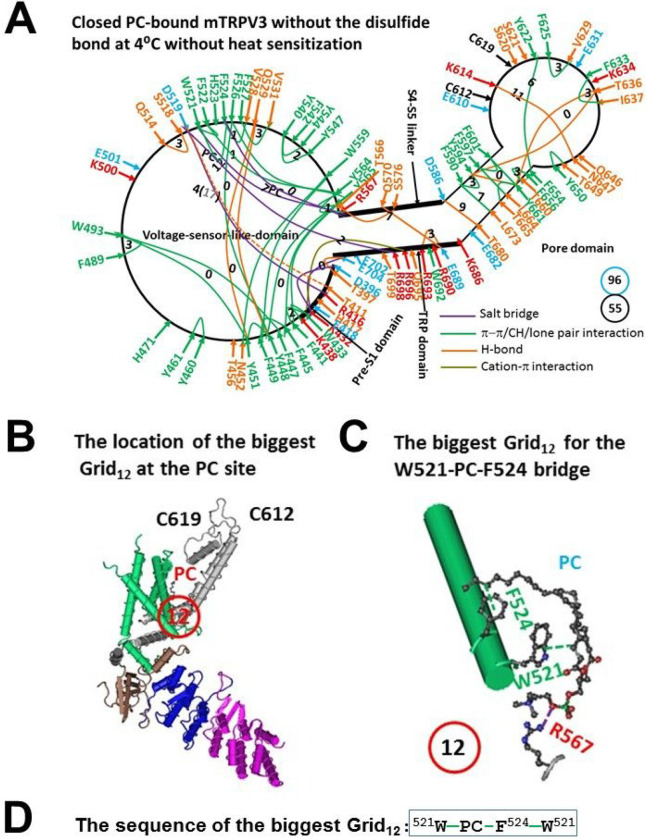
The grid-like non-covalently interacting mesh network along the gating pathway of PC-bound reduced mTRPV3 in the closed state at 4 °C without heat sensitization. **A,** The topological grids in the systemic fluidic grid-like mesh network. The cryo-EM structure of one subunit in reduced and closed mTRPV3 with PC bound in MSP2N at 4 °C (PDB ID, 6LGP) was used for the model. The pore domain, the S4-S5 linker, the TRP domain, the VSLD and the pre-S1 domain are indicated in black. Salt bridges, p interactions, and H-bonds between pairing amino acid side chains along the gating pathway from D396 to K705 are marked in purple, green, and orange, respectively. The grid sizes required to control the relevant non-covalent crosslinking interactions were calculated with graph theory and labeled in black. The total grid sizes and grid size-controlled non-covalent crosslinking interactions along the gating pathway are shown in the blue and black circles, respectively. The T411-D519 H-bond, which is marked in a dashed line, may be disrupted by the insertion of valine or serine at position 412. **B,** The location of the biggest Grid_12_ is marked in a red circle. **C,** The structure of the biggest Grid_12_ with a 12-atom size at the PC site to control the W521-PC-F524 bridge. **D,** The sequence of the biggest Grid_12_ to control the W521-PC-F524 bridge in a blue box.

In the absence of the C612-C619 disulfide bond, reduced mTRPV3 had some different noncovalent interactions to form the systematic grid-like non-covalent interaction mesh network at 4°C when compared with the closed and oxidized one at 42°C after heat sensitization (Figs. 4A & 5A, Table S5) [32]. In the pore domain, after the E610-K614 salt bridge and the E610-N647 and S621-Q646 and E682-K686 H-bonds were disrupted, the F625-V629 and F633-I637 and T649-Y650 π interactions and the Y594-Y661 H-bond emerged (Fig. 5A).

When this conformational change extended to the S4-S5 linker/TRP interface, the R567-T699 H-bond and the R698-E702 salt bridge were broken but the Q570-E689 H-bond replaced the Q570-W692 CH- π interaction. As a consequence, along with the T566-S576 H-bond in the S4-S5 linker/VSLD interface, D519 H-bonded with T411 in the VSLD/pre S1 interface and formed an additional salt bridge with R698 in the VSLD/TRP interface (Fig. 5A).

When this conformational change extended to the VSLD, the T456-W559 H-bond was broken, the H471-Y540/Y547 π interaction network changed to the Y448/Y551-Q529 H-bonds, the π interaction moved from F542-Y544 to Y540-Y547, and the H-bond shifted from K500-E501 to Q514-S518. When the PC bridge moved from W521-PC-Q695 to W521-PC-F524/R567 (Figs. 4B & 5B), in addition to a salt bridge between R567 and the PC lipid and the CH-π interactions of the PC lipid with W521 and F524, Y564 formed a CH-π interaction with R567 (Fig. 5A).

By all account, the disruption of the C612-C619 disulfide bond brought about the biggest Grid_12_ at the vanilloid PC site (Fig. 5B). When two equivalent H-bonds governed the 12-atom path from W521 to PC to F524 (Figs. 5C–D), the predicted melting temperature was about 50°C (Table 1), which was close to the initial experimental T_th_ 52°C for TRPV3 opening [19]. On the other hand, when compared with oxidized mTRPV3 in both closed and open states, only four H-bonds and 26 π interactions were conserved, and two new salt bridges and four new H-bonds and six new π interactions were added (Tables S3, S4 and S5). As the total non-covalent interactions and grid sizes along the gating pathway from D396 to K705 were 55 and 96, respectively (Fig. 5A), the systemic thermal instability (T_i_) was 1.75 (Table 1). When the same open state as shown in the oxidized and PC-free mTRPV3 was employed (Fig. 4A), the melting of the W521-PC-F524 bridge in Grid_12_ would produce the calculated Ω_10_ ranging from 8.76 to 58.5 with a mean value 18.3, which was approximate with the experimental Q_10_ (16.4–22.6) (Table 1) [19]. Thus, the initial high T_th_ and Q_10_ of mTRPV3 upon the brief heat stimulation may result from the gating transition from the reduced and closed state to the open and oxidized one.

## Discussion

Thermo-sensitive TRPV3 is characterized as the use-dependent heat sensitization. Although several cryo-EM structures of mTRPV3 are available in different gating and redox states and at various temperatures, the specific structural motifs responsible for this use-dependent heat sensitization are still missing. This computational study first demonstrated that the calculated melting temperature threshold $\left(T_{m}\right)$ of the biggest grid along the gating pathway in mTRPV3 was comparable to not only the structural $T_{m}$ but also the functional activation threshold T_th_ of mTRPV3. It further confirmed that the functional thermo-sensitivity Q_10_ was also comparable to the grid-based structural thermo-sensitivity Ω_10_. Finally, the grid-based systematic thermal instability values of mTRPV3 in different redox- and lipid-dependent gating states were also compared with each other to establish the energetic relationship of different gating states. Taken as a whole, three gating states were completely identified to account for the use-dependent heat sensitization of TRPV3.

First, it was further confirmed that the biggest grid may employ its size and strength to determine the melting temperature threshold ${(T}_{m})$ of TRPV3. At a given salt concentration (150 mM NaCl), for reduced and sensitized mTRPV3-Y564A, when 1.0 equivalent H-bond sealed the biggest Grid_13_, it had a calculated $T_{m}$ of about 38°C in the closed state. In agreement with this prediction, the D396-K432 salt bridge in this Grid_13_ was broken in the open state at 37°C (Figs. 1A and 2A, Table 1) [22]. For oxidized and sensitized mTRPV3 in the closed state, when two equivalent H-bonds sealed the biggest Grid_17_, it had a calculated $T_{m}$ 40°C, and the R416-D519 salt bridge was also disrupted at 42°C in the open state (Figs. 3A & 4A, Table 1) [23].

Second, if the functional temperature threshold T_th_ for activation of mTRPV3 is controlled by the melting temperature threshold ${(T}_{m})$ of the biggest grid along the gating pathway, the calculated $T_{m}$ should be comparable to the measured threshold T_th_. In accordance with this prediction, reduced mTRPV3 in the closed state had a T_th_ around 52°C which was close to the calculated $T_{m}$ 50°C of the biggest Grid_12_ at the vanilloid PC site (Fig. 5A, Table 1) [19]. Once oxidized, mTRPV3 had a low calculated $T_{m}$ of 40°C (Table 1). Since the tunable distance between R416 in the pre-S1 domain and D519 in the VSLD, the R416-D519 salt bridge may change the strength which ranged from 0.5 to 2.0 equivalent H-bonds, That may account for the warm activation of mTRPV3 in a range from around 25–40°C [5–6, 19, 33]. Hence, the activation threshold T_th_ may be governed by the melting of the biggest grid via the adjustable R416-D519 salt bridge along the gating pathway. Since the calculated $T_{m}$ values of the biggest Grid_9_ and Grid_11_ in the open states of oxidized mTRPV3 and reduced mTRPV3-Y564A were about 56°C and 57°C, respectively (Figs. 2A & 4A, Table 1), the biggest grids along the gating pathway may be responsible for the optimal activity temperature range of the mTRPV3 bio-thermometer [19].

Third, increased temperature has been reported to accelerate the dissociation rate $\left(k_{d}\right)$ of enthalpy-driven non-covalent interaction in a biophysical network but to slow down $k_{d}$ of entropy-driven crosslinks to a different extent [34]. In this study, an increase in the opening rate or the open probability $\left(P_{o}\right)$ of TRPV3 has been observed with raised temperatures [23]. If the temperature threshold for TRPV3 opening is governed by a rate-limiting single step to disrupt a non-covalent interaction in the biggest grid along the gating pathway, TRPV3 opening would be initially enthalpy-driven (△H<0). In this case, when thermo-gated TRPV3 opens from a closed state within 10°C, the functional thermo-sensitivity (Q_10_) should be comparable to the calculated systematic structural thermo-sensitivity Ω_10_ because they both factually reflect the change of the total chemical potentials of all the grids upon the alteration of the total enthalpy included in the non-covalent interactions along the same gating pathway from D396 to K705. In agreement with this proposal, if wild-type mTRPV3 had the same open and oxidized state, the calculated mean Ω_10_ of reduced mTRPV3 would be 18.3, which was close to the measured Q_10_ (16.4–22.6) (Table 1) [19]. For oxidized and sensitized mTRPV3, the calculated mean Ω_10_ was 4.12, which was similar to the measured Q_10_ (1.9–3.1) (Table 1) [19]. For the reduced mTRPV3-Y564A mutant, the calculated mean Ω_10_ was 1.48, which was near to the measured Q_10_ 1.21 (Table 1) [22]. Thereafter, the functional thermo-sensitivity Q_10_ may be governed by the grid-based systematic structural thermo-sensitivity Ω_10_ as defined. In this regard, when the intensity of a non-covalent interaction was in the range from 0.5 to 3 kJ/mol, the resultant Ω_10_ ranges from the minimum to the maximum may be theoretically calculated as 8.76–58.5 for reduced and closed mTRPV3, 1.88–14.3 for sensitized and oxidized mTRPV3, and 0.76–4.3 for reduced and sensitized mTRPV3-Y564A (Table 1).

**Figure 6:**
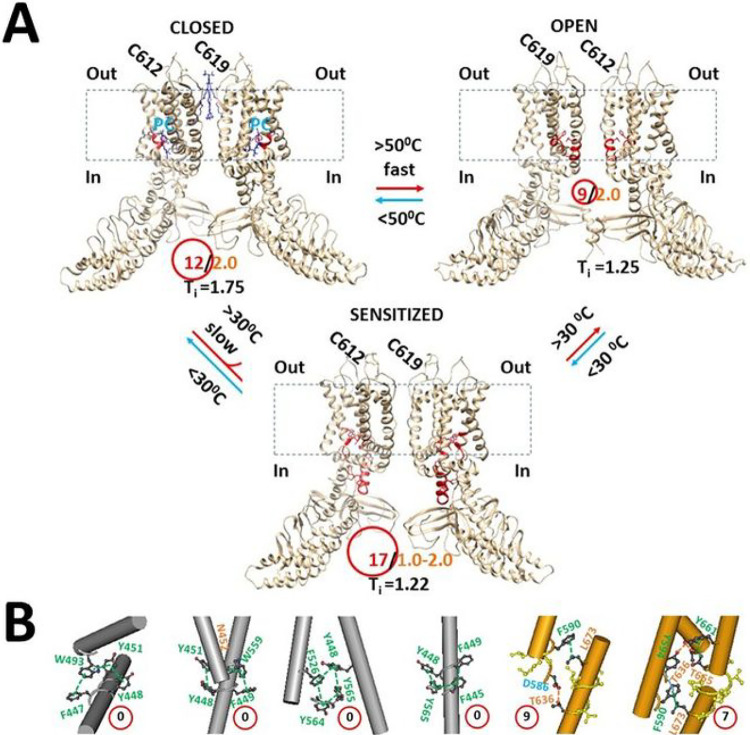
The tentative model for the use-dependent heat sensitization of thermo-gated TRPV3. **A,** The homo-tetrameric cryo-EM structures of mTRPV3 in the reduced and closed state (PDB ID: 6LGP), the sensitized and oxidized (PDB ID: 7MIN), and the open and oxidized state (PDB ID: 7MIO) were used for the model. For a convenient view, only two opposite subunits are shown. The dashed rectangle is the membrane area. In the absence of the C612-C619 disulfide bond, reduced mTRPV3 is open with a high Q_10_ (16.4–22.6) upon the short heat stimulus above 50 °C to melt the biggest Grid_12_, a thermo-active ring (red), at the vanilloid PC site and then to release PC from the vanilloid site. Meanwhile, C612 is close to C619 enough to form a disulfide bond. When the temperature decreases below 40 °C, oxidized and PC-free mTRPV3 is closed with the biggest Grid_17_, another thermo-active ring (red), in the VSLD to decrease the threshold from 50 °C to 30–40 °C. Upon the second short heat stimulus, the sensitized and oxidized mTRPV3 has a Q_10_ as low as 1.9–3.1. However, the long and slow warm stimulation above 30 °C can oxidize mTRPV3 to decrease the threshold from 50 °C to 30 °C in favor of the release of the PC lipid from the vanillloid site for channel opening but with a low Q_10_ (1.66). **B,** The proposed smaller thermo-stable anchor grids against which mTRPV3 opens above the temperature thresholds (PDB ID, 7MIO). The grid sizes are shown in the red circles.

Taken together, it is proposed that reduced mTRPV3 may start the first activation above the calculated $T_{m}$ 50°C upon the fast heat stimulation. Once the channel is opened, it is oxidized to form the C612-C619 disulfide bond so that the functional thermo-sensitivity Q_10_ (16.4–22.6) can keep consistent with the calculated Ω_10_ (18.3) (Fig. 6A, Table 1). It is further proposed that when the temperature declines, oxidized but sensitized mTRPV3 may decrease a T_th_ to 30–40°C and Q_10_ to 1.9–3.1 as a result of the formation of the C612-C619 disulfide-bond (Fig. 6A, Table 1) [19]. In this way, the lower threshold 30–40°C may increase the open probability in response to the same temperature jump from 32°C to 59°C so that mTRPV3 activation exhibits the use-dependent sensitization upon successive heat stimuli [19]. In direct line with this use-dependent sensitization, the grid-based systemic thermal instability ${(T}_{i})$ in the closed state was 1.75 for reduced mTRPV3, and slightly decreased to 1.22 when oxidized by heat sensitization in favor of the use-dependent heat-sensitization during channel opening with a similar $T_{i}$ of 1.25 (Table 1). In contrast, the Y564A mutation increased the grid-based systemic thermal instability ${(T}_{i})$ from 1.75 to 2.03 for the sensitized but closed state and 1.90 for the open state (Table 1). In other words, the Y564A mutation may increase the systemic thermal instability in favor of spontaneous channel opening [22].

On the other hand, when reduced or Cys-less mTRPV3 is exposed to the long and slow heat stimulation, the channel can be activated above a threshold T_th_ 30°C [33]. Therefore, it is also possible that either the formation of the C612-C619 disulfide bond by air oxidation or the Cys-less mutation may increase the length of the R416-D519 salt bridge to account for the declined T_th_ of 30°C so that the PC lipid could be released from the vanilloid site for channel opening (Fig. 6A, Table 1). When the insertion of valine at position 412 disrupts the T411-D519 salt bridge and the related smaller Grid_4_ via the shortest path from T411 to R416 and D519 and then back to T411 (Figs. 5A, 6B), the same biggest Grid_17_ as shown in oxidized but closed mTRPV3 may be followed in the VSLD/pre-S1 interface in favor of the release of the vanilloid site lipid for channel opening below 40°C in response to the first fast heat stimulus (Figs. 3A, 5A, Table 1). That may be why the insertion of valine at 412 removes the use-dependent sensitization upon repeated heat stimuli [19]. In support of this proposal, when the Y564A mutation release the PC lipid from the vanilloid site, it also had a low calculated $T_{m}$ (38°C) and Ω_10_ (1.48) to keep consistent with the low threshold (< 37°C) and the low Q_10_ (1.21), respectively (Fig. 1–2, Table 1) [22].

In any way, several smaller anchor grids in the pore domain may be important to stablize the common open state in favor of high heat efficacy. In the pore domain, the first was Grid_7_ with the shortest path from F590 to Y594, T636, Y661, T665, L673 and back to F590, and the second was Grid_9_ with the shortest path from D586 to F590, L673 and T680 and then back to D586 (Figs. 4A, 4C, 6B). It has been reported that the T636S mutation decreases the temperature threshold [33], and the mutation N643S, I644S, N647Y, L657I, Y661C or T680A is actually less sensitive to heat or slows down the activation rate [30, 32, 35–36]. Therefore, it is possible that these mutations may affect the thermostability of these smaller anchor grids in the pore domain.

In contrast, four smallest grids with a zero-residue size in the VSLD may form a basic stable backbone anchor system for mTRPV3 activation or a fuse group to keep a low systemic thermal instability (Figs. 3– 5, 6B): the first Grid_0_ via the shortest path from F447 to W493, Y451, Y448 and back to F447; the second Grid_0_ via the shortest path from Y448 to Y451, N452, W559, F449 and back to Y448; the third Grid_0_ via the shortest path from Y448 to F526, Y564, Y565 and back to Y448; the forth Grid_0_ via the shortest path from F445, Y448, F449 and back to F445.

Further experiments may be required to test if non-covalent interactions in other grids than anchors are essential for heat-evoked TRPV3 opening. For example, the D519-R416 salt bridge, the H417/E418-R690 and D519-R567 and S620-Q646 and Q529-Y448/Y451 H-bonds, the F441-Y565 and H471-Y540/Y547 and Y622/Q646-F654 π−π interactions, and the Q570-W692-R696-W433-K438 cation/CH-π interactions (Figs. 3A, 4A, 5A).

## CONCLUSION

In this computational study, a graphical grid thermodynamic model has bridged crystallographic static conformations with electrophysiological dynamic findings together by using graph theory in atomic details. Once the thermal rings in the systematic fluidic grid-like mesh network of non-covalent interactions along the gating pathway were tested and identified as key deterministic structural factors or motifs for thermo-gated mTRPV3, three gating states could be in turn established to account for the use-dependent heat sensitization of TRPV3. Accordingly, this model can be used to predict the thermal stability and activity of cellular biological macromolecules including membrane proteins once high-resolution 3D structural data are available.

## METHODS

### Data mining resources

In this *in silico* study, two groups of the temperature-dependent cryo-EM structures of mTRPV3 in different gating and redox states were analyzed by graph theory to abstract the structural bioinformation for the use-dependent temperature thresholds and sensitivity. One included reduced and sensitized mTRPV3-Y564A at 37°C in the detergent (PDB ID, 6PVO, model resolution = 5.18 Å) and reduced and open mTRPV3-Y564A (PDB ID, 6PVP, model resolution = 4.4 Å) at 37°C in the detergent [22]; The other covered oxidized and sensitized WT mTRPV3 in cNW11 at 42°C (PDB ID, 7MIN, model resolution = 3.09 Å), oxidized and open WT mTRPV3 in cNW11 at 42°C (PDB ID, 7MIO, model resolution = 3.48 Å) [23], and reduced and closed WT mTRPV3 in MSP2N2 at 4°C (PDB ID, 6LGP, model resolution = 3.31 Å) [32].

### Standards for non-covalent interactions

In order to secure that results could be reproduced with a high sensitivity, the same strict standard definition as described previously as well as structure visualization software, UCSF Chimera, was exploited to identify stereo- or regio-selective inter-domain diagonal and intra-domain lateral non-covalent interactions in the 3D strcutres of mTRPV3 (Tables S1-S5) [27–29]. They included salt-bridges, CH/cation/lone pair/π−π interactions and H-bonds along the gating pathway from D396 to K705 in mTRPV3 with or without the Y564A mutation. Notably, although the hydrophobic effect and residue hydrophobicity are necessary to drive protein folding, their effects on protein stabilization may be rather marginal [37–38].

### Preparation of topological grid maps by using graph theory

The identified non-covalent interactions were geometrically mapped as edges along with marked node arrows to represent the positions of the linked residues in the systematic fluidic mesh network according to the same protocol as previously described [27–29]. All the grids were then covered in this network after their ring sizes were constrained as the minimal number of the total free side chains of residues or atoms in the bound lipid that did not participate in any non-covalent interction in a grid. The size constraint was completed by using graph theory and the Floyd-Warshall algorithm to calculate the shortest return path from one end of a non-covalent interaction to the start because the direct path from the start to the end was zero. [39]. For example, in the intra-subunit grid-like biochemical reaction mesh network of Fig. 3A, a direct path length from E610 and N647 was zero because of an H-bond between them. However, there was another shortest return path from N647 to K614 and back to E610 via the N647-K614 H-bond and the K614-E610 salt bridge in this grid. Therefore, the grid size was zero. Once all the grid sizes were available, only the uncommon sizes were marked in black, and a grid with an x-residue or atom size was denoted as Gridx. When the total number of all noncovalent interactions and grid sizes along the gating pathway were calculated, they were displayed in black and blue circles beside the mesh network map, respectively, for the calculations of the systematic thermal instability and the structural temperature sensitivity.

### Calculation of the temperature threshold of mTRPV3

A DNA hairpin thermo-sensor with a 20-base loop and two G-C base pairs in the stem has a start control melting temperature threshold $\left(T_{m}\right)$ of 34°C to initiate thermal unfolding of the hairpin loop. When an additional G-C base or five additional bases are included in the hairpin, the $T_{m}$ is increased by 10°C [26]. In a similar way, when a single polypeptide chain in protein carries out rate-limiting thermal unfolding of the thermal rings from the biggest grid to the smallest grid, the $T_{m}$ of thermal unfolding of the given grid along the chain was calculated by using the following equation as described previously [27–29]:

(1) $$T_{m}\left({\;}^{\circ }C\right)=34+(n-2)\times 10+\left(20-S_{max}\right)\times 2$$

where, n is the total number of the grid size-controlled H-bonds equivalent to non-covalent interactions in the given grid, and $S_{max}$ is the size of the given grid. In this regard, the more grid’s heat capacity will be expected with the decreased grid size or the increased equivalent H-bonds.

### Calculation of the systemic thermal instability $\left(T_{i}\right)$

On the other hand, the $T_{m}$ of the DNA hairpin will be always increased by the more G-C base pairs in the stem or the shorter the poly-A loop [26]. Thus, the grid-based systemic thermal instability $\left(T_{i}\right)$ along the single polypeptide chain was reasonably defined using the following equation as described previously [27–29]:

(2) $$T_{i}=S/N$$

where, S is the total grid sizes and N is the total non-covalent interactions along the gating pathway of one subunit in a gating state. Usually, the lower $T_{i}$, the less the conformational entropy in the system.

### Calculation of the systematic temperature sensitivity of mTRPV3

For initial enthalpy-driven TRPV3 opening upon decyclization of the biggest grid (ΔH<0), if a thermosensitive TRPV3 channel changes from a fully closed state to a fully open state within a temperature range ΔT, and if the chemical potential of a grid is defined as the maximal potential for equivalent residues in the grid to form a tight and ideal β -hairpin with the smallest loop via non-covalent interactions [40], the grid-based systematic structural thermo-sensitivity $\left(\Omega_{\Delta T}\right)$ of a single ion channel can be defined and calculated using the following equations:

(3) $$\Omega_{\Delta T}={\left[\left(S_{c^{-}}S_{o}\right)E/2\right]}^{\left(Hc/Ho\right)}={\left[\left(S_{c}-S_{o}\right)E/2\right]}^{\left[(ENc)/(ENo)\right]}={\left[\left(S_{c}-S_{o}\right)E/2\right]}^{\left(Nc/No\right)}$$

where, in the closed and open states along the same gating pathway of one subunit, $H_{c}$ and $H_{o}$ are the total enthalpy included in non-covalent interactions, respectively; $N_{c}$ and $N_{o}$ are the total non-covalent interactions, respectively; $S_{c}$ and $S_{o}$ are the total grid sizes, respectively. E is the energy intensity of a non-covalent interaction in a range of 0.5–3 kJ/mol. Usually, E is 1 kJ/mol. Thus, $\Omega_{\Delta T}$ actually mirrors a heat-evoked change in the total chemical potential of all the grids upon a heat-induced change in the total enthalpy included in non-covalent interactions from a closed state to an open state along the same gating pathway of one subunit.

When $\Delta T=10^{\circ }C$, Ω_10_ could be comparable to the functional thermo-sensitivity (Q_10_) of a single ion channel. Q_10_ was defined and calculated using the following equation:

(4) $$Q_{10}={\left(X_{2}/X_{1}\right)}^{10/(T2-T1)}$$

where, $X_{1}$ and $X_{2}$ are open probability $\left(P_{o}\right)$ values or reaction rates obtained at temperatures T1 and T2 (measured in kelvin), respectively.

## Figures and Tables

**Table 1:** The grid thermodynamic model-based new parameters of the mTRPV3 bio-thermometer along the gating pathway from D396 to K705

**Construct**	**WT mTRPV3**			**mTRPV3-Y564A**	
**PDB ID**	6LGP	7MIO	7MIN	6PVP	6PVO
**Lipid PC at the vanilloid site**	bound	free	bound	free	free
**Redox state**	reduced	oxidized	oxidized	reduced	reduced
**Lipid environment**	MSP2N2	cNW11	cNW11	detergent	
**Sampling temperature, °C**	**4**	**42**	**42**	**37**	**37**
**Gating state**	**Closed ↔ Open ↔ Sensitized**			**Open ↔Sensitized**	
**# of the biggest grid**	Grid_12_	Grid_9_	Grid_17_	Grid_n_	Grid_13_
**Biggest grid size (S_max_)**	12	9	17	11	13
**Equivalent H-bonds in S_max_**	2.0	2.0	2.0	2.0	1.0
**Total non-covalent interactions**	55	52	59	39	38
**Total grid sizes, a.a**	96	65	72	74	77
**Calculated T_m_, °C**	**50**	56	**40**	52	**38**
**Measured T_m_, °C**			**42**		**37**
**Measured T_th_, °C**	**52**	**57**	**32–39**		
**Systemic thermal instability (T_i_)**	**1.75**	**1.25**	**1.22**	1.90	**2.03**
Calculated Ω_10, min_ at E_min_=0.5 kJ/mol	8.76		1.88		0.76
**Calculated Ω_10, mean_ at E_mean_=1.0 kJ/mol**	**18.3**		**4.12**		**1.48**
Calculated Ω _10, max_ at E_max_=3.0 kJ/mol	58.5		14.3		4.30
**Measured Ω _10_,**	**16.4–22.6**		**1.9–3.1**		**1.21**
**Ref. for measured T_th_ or Q_10_**	[19]	[19]	[19]		[19]

## Data Availability

All data generated or analysed during this study are included in this published article and Supplementary Information.

## Conventions and abbreviations

ARD: ankyrin repeat domain
cryo-EM: cryo-electron microscopy
$T_{m}$,: melting temperature
PC: phosphatidylcholine
T_th_: temperature threshold
TMD: transmembrane domain
TRP: transient receptor potential
TRPA: TRP ankyrin
TRPC: TRP canonical
TRPVi: TRP vanilloid i
TRPV3: TRP vanilloid-3
rTRPV1: rat TRPV1
mTRPV3: mouse TRPV3
TRPMi: TRP melastatin i
TRPM8: TRP melastatin 8
VSLD: voltage-sensor-like domain
WT: wild-type
